# Synergistic Inhibition of Plantaricin E/F and Lactic Acid Against *Aeromonas hydrophila* LPL-1 Reveals the Novel Potential of Class IIb Bacteriocin

**DOI:** 10.3389/fmicb.2022.774184

**Published:** 2022-02-15

**Authors:** Yang Wang, Yunlu Wei, Nan Shang, Pinglan Li

**Affiliations:** ^1^Beijing Laboratory for Food Quality and Safety, College of Food Science and Nutritional Engineering, China Agricultural University, Beijing, China; ^2^Tianjin Key Laboratory of Aqua-Ecology and Aquaculture College of Fisheries, Tianjin Agricultural University, Tianjin, China; ^3^College of Engineering, China Agricultural University, Beijing, China; ^4^Key Laboratory of Precision Nutrition and Food Quality, Department of Nutrition and Health, China Agricultural University, Beijing, China; ^5^School of Life Science and Engineering, Southwest University of Science and Technology, Mianyang, China

**Keywords:** lactic acid, bacteriocin, Gram-negative bacteria, lipopolysaccharide, inhibition

## Abstract

Plantaricin E/F (PlnEF) is a pair of two-component class IIb bacteriocin produced by lactic acid bacteria. PlnEF commonly displays potent antimicrobial activity against certain Gram-positive organisms. In this study, we investigated the synergistic activity of PlnEF combined with lactic acid against Gram-negative food and aquaculture potential pathogen *Aeromonas hydrophila* LPL-1, which is naturally resistant to PlnEF. We applied SDS-PAGE, wavelength-scanning, laser confocal microscopy, flow cytometer, scanning electron microscopy (SEM), transmission electron microscopy (TEM), and two-dimensional electrophoresis to investigate their synergistic inhibitory activities. The results showed that L-lactic acid drove the release of LPS from *A. hydrophila*, making it possible for PlnEF to contact the inner cell membrane of *A. hydrophila*. Besides, co-treatment of lactic acid and PlnEF caused severe morphological and intracellular changes of *A. hydrophila*, including blebs on the cell surface, abnormal cell elongation, inner membrane disruption, pore-forming through the outer and inner membrane, coagulation of the cytoplasm, and structural transformation of DNA. Protein profile analysis revealed that combined treatment of lactic acid and PlnEF inhibited the energy metabolism, protein synthesis, protein folding, and DNA replication in *A. hydrophila*. These findings proved that PlnEF combined with lactic acid was efficient against *A. hydrophila* and shed light on bacteriocin’s potential and a new inhibition mechanism against *A. hydrophila*.

**Importance:**

Bacteriocins and their producing strains are increasingly used to substitute artificial preservatives and antibiotics in the food and aquaculture industries. However, the bacteriocins produced by lactic acid bacteria are efficient to mainly Gram-positive bacteria. Our paper had demonstrated the antimicrobial activity of class IIb bacteriocin against potential Gram-negative pathogen, *A. hydrophila* LPL-1, when combined with lactic acid. The results could refresh our knowledge about the potential of class IIb bacteriocins produced by lactic acid bacteria.

## Introduction

Lactic acid bacteria (LAB) are the most commonly used probiotics that can inhibit or kill various Gram-positive and Gram-negative pathogens. The main antibacterial substances produced by LAB include organic acid (Özcelik et al., 2016), bacteriocin (Mokoena, 2017), H_2_O_2_ (Dashe et al., 2020), enzymes, acetoin, acetaldehyde, etc. (Moradi et al., 2020).

Bacteriocins produced by lactic acid bacteria are ribosomally synthesized cationic peptides with or without post-translational modification, which can be grouped into different classes (Cotter et al., 2005; García et al., 2010; Alvarez-Sieiro et al., 2016). Among them, the well-studied class I and class II bacteriocins have been used in the food industry (nisin and pediocin PA-1) (Gharsallaoui et al., 2016; Santos et al., 2018; Ibarra-Sánchez et al., 2020; Anumudu et al., 2021) and shown a great potential as an alternative to antibiotics (Bonhi and Imran, 2019; Lopetuso et al., 2019; Fu and Kapila, 2021). The mode of action for class I bacteriocins (e.g., nisin) is inhibition of peptidoglycan synthesis and forming pores in the cell membrane. Class II bacteriocins can form pores in the cell membrane (Cotter et al., 2013). The majority of class I and class II bacteriocins produced by lactic acid bacteria are commonly known to inhibit Gram-positive pathogens but have limited inhibitory effects against Gram-negative bacteria (Cotter et al., 2005; García et al., 2010; Cui et al., 2021). However, studies have found several bacteriocins produced by LAB strains that display a stronger antagonism to Gram-negative bacteria (Riley and Wertz, 2002; Todorov and Dicks, 2005; Rumjuankiat et al., 2015; Sahoo et al., 2015; Yang et al., 2015; Kumariya et al., 2019; Sheoran and Tiwari, 2019; Haghighatafshar et al., 2021). Gram-negative bacteria are resistant to many antimicrobial substances due to the impermeability of their outer membrane (OM) (Christaki et al., 2020). Disruption of the OM barrier allows for the entry of otherwise inactive antimicrobials into Gram-negative pathogens, thus sensitizing the bacteria (Savage, 2001; Ciepluch et al., 2019; MacNair and Brown, 2020). It is proposed that the OM perturbants or permeabilizers such as lactic acid (Kalchayanand et al., 1992; Nykänen et al., 1998), chelating agent (Alakomi et al., 2003; Martin-Visscher et al., 2011), polycations, hydrophobic antibiotics, detergents, lysozyme, polyanionic polyethyleneimine (Helander et al., 1997), polymyxin B (Chi and Holo, 2018), protamine, etc. (Johansen et al., 1997), and some bacteriocins (Sheoran and Tiwari, 2021) could broaden the antibacterial spectrum of bacteriocins, thus potentiating Gram-negative bacteria inhibition. Among the reported OM permeabilizers, lactic acid is a promising metabolite. As the most common metabolite of LAB, lactic acid has been reported to be an outer membrane permeability agent leading to the release of outer membrane LPS in Gram-negative bacteria (Alakomi et al., 2000; Wang et al., 2020), thus increasing the sensitivity of *A. hydrophila* to class IIa bacteriocin, pediocin PA-1 (Wang et al., 2020). The lipopolysaccharide (LPS) is enriched in the outer membrane of Gram-negative bacteria, providing a natural barrier against many antibotics (Cetuk et al., 2021) and the bacteriocins that are mainly effective against Gram-positive bacteria (Kalchayanand et al., 1992; Gao et al., 1999; Osmanağaoğlu, 2005). Due to the LPS barrier’s damage, lactic acid-treated Gram-negative bacteria could be more sensitive to bacteriocins. Nykänen et al. has elucidated the synergistic potential of class I bacteriocin-nisin and lactic acid against Gram-negative pathogen *Pseudomonas fluorescens* and *Pseudomonas aeruginosa* ATCC9721 (Nykänen et al., 1998). Kalchayanand et al. found that acid stress (40% lactic acid, 16% propionic acid, and 16% acetic acid in water) increases the sensitivity of *Yersinia enterocolitica* Y7P and *Pseudomonas fluorescens* PF2 to nisin and pediocin AcH (Kalchayanand et al., 1992). We have proved the synergistic activity of class IIa bacteriocin-pediocin PA-1 and lactic acid against *A. hydrophila* and provided a potential mechanism of their synergistic inhibitory mechanism. L-lactic acid released the outer membrane LPS, making it possible for pediocin PA-1 to contact the plasma membrane of *A. hydrophila*, resulting in the dissipation of proton-motive force in the inner membrane and cell death (Wang et al., 2020). However, there were no reports about the combined use of class IIb bacteriocins with lactic acid. Class IIb bacteriocins possess a different antibacterial spectrum (Wu et al., 2021), stability, receptors, and action of mode compared with class I and class IIa bacteriocins (Cotter et al., 2013). They are reported to be alternative antimicrobial peptides in food preservation (Abdulhussain Kareem and Razavi, 2020). Besides, their producing strains, including *Lactiplantibacillus plantarum* (former *Lactobacillus plantarum*), *Enterococcus faecalis* (Maldonado-Barragán et al., 2009), *Lactococcus lactis* (Zendo et al., 2006), identified as potential probiotics, are promising for the-insuit application of bacteriocin producing strains in food, agriculture or pharmaceutical field (Yin et al., 2018). Therefore, it is urgent to figure out the synergistic activity of lactic acid and class IIb bacteriocin and its underlying mechanism for better application of class IIb bacteriocin and their producing strains in Gram-negative pathogen control. Class IIb bacteriocins are ribosomally synthesized, unmodified, two-peptide bacteriocins. The antimicrobial activities of class IIb bacteriocin rely on the complementary action of the two peptides (Ekblad et al., 2016). So far, more than 15 pairs of two peptide bacteriocins have been identified (Nissen-Meyer et al., 2010). Among them, plantaricin E/F showed efficient inhibitory activity on Gram-positive bacteria by causing cell membrane damage, resulting in the dissipation of cell proton motive force and finally causing cell death (Zhang et al., 2016).

*Aeromonas hydrophila* is an important Gram-negative pathogen associated with various human diseases (Ottaviani et al., 2011; Igbinosa et al., 2012; Kali et al., 2016) and aquatic animal diseases (Mzula et al., 2019; Anjur et al., 2021). *Furthermore, A. hydrophlia* are reported to be resistant to many antibiotics (Stratev and Odeyemi, 2016). And they are causing significant economic loss worldwide and are considerable threats to food safety and aquaculture (Praveen et al., 2016). Therefore, controlling *A. hydrophila* is necessary for aquaculture and food safety (Daskalov, 2006; Pal, 2018). The potential pathogen *A. hydrophila* LPL-1 was originally isolated from the spoiled sturgeon flesh sample in Beijing in 2013. The spoilage role of strain *A. hydrophila* LPL-1 was confirmed with fresh sturgeon fish flesh. With inoculation of 10^6^ CFU/g of *A. hydrophila* LPL-1, the fish flesh was seriously spoiled after two days of refrigerator storage. Meanwhile, *A. hydrophila* LPL-1 also caused 100% mortality in zebrafish after one day of the soaking challenge with 1 × 10^9^CFU/mL viable bacteria (unpublished research).

In this study, we investigated the synergistic inhibitory effect of PlnEF and lactic acid against *A. hydrophila* and its underlying mechanisms. A potential pathogenic strain *A. hydrophila* LPL-1 was used to evaluate the inhibition effect, the damage of cell structure, and alternation in cellular protein profile.

## Materials and Methods

### Strains and Growth Conditions

The strain *A. hydrophila* LPL-1 was originally isolated from spoiled sturgeon in our lab and identified its species by 16S ribosomal RNA gene sequencing and whole-genome sequencing (WGS) (GenBank, BioProject ID PRJNA767215)^1^. *A. hydrophila* LPL-1 shares 3,795 genes, or 94.44% genes with the representative of *Aeromonas hydrophila* ATCC 7966 (Supplementary Figure 1). The bacteria cultured in Luria-Bertani (LB) liquid medium for 24 h was collected by centrifugation (1600 g, 5 min, 4°C), resuspended with sterile skim milk, and freeze-dried. The bacterial powder was stored at −80°C and was routinely cultured in LB broth at 30°C under aerobic conditions.

### Bacteriocin Synthesis and Fluorescent Label

The mature peptides of plantaricin E (PlnE: FNRDGY NFGKSVRHVVDAIGSVAGIRGILKSIR) and plantaricin F (PlnF: VFHAYSARGVRNNYKSAVGPADWVISAVRGFIHG) were synthesized using the solid-phase synthesis method by Gill biochemical Shanghai Co., LTD., China (purity (HPLC) > 95%, Supplementary Figure 2). PlnE was fluorescently labeled by fluorescein isothiocyanate (FITC) as follows: FITC (2 eq.) (resolved in Pyridine (2 eq.) and N, N-Diisopropylethylamine (DIPEA 2 eq.) was mixed with the crude peptide for 1 h. FITC-labeled PlnE was subsequently purified to over 95% chromatographic homogeneity by reverse-phase high-performance liquid chromatography and confirmed by mass spectrometry analysis.

### Antibacterial Activity of PlnEF Against *Aeromonas hydrophila*

About 1.0 × 10^7^ CFU/mL (confirmed by plate count method) *A. hydrophila* LPL-1 was inoculated in LB broth with 10 mM L-lactic acid, 10 mM L-lactic acid with different levels of PlnEF (0.5, 2, 5, 10, 25 μM), and an equal volume of distilled water (as control). All the samples were cultured at 30°C for 12 h in a 96-well plate and subsequently analyzed for OD_600_ using a Multi-function microplate reader (Thermo Scientific Varioskan Flash). The minimum inhibitory concentration (MIC90) values were defined as the lowest concentration of PlnEF at which the growth of bacteria in 90% of the microplates was inhibited.

The bactericidal activity was measured by propidium iodide (PI) staining. Briefly, *A. hydrophila* (0.8-1.0 × 10^8^ CFU/mL, confirmed by plate count method) were collected and resuspended in saline (as control), saline with 10 mM L-lactic acid, saline with 10 mM L-lactic acid combined with 25 μM PlnEF, respectively, and incubated at 30°C for 2, 4, 6, 8 h. After that, the cells were washed with sterile saline to remove the antimicrobial substances, and other substances interfered with PI. Bacterial cells resuspended with saline were incubated with 10 μM PI for 1 h at 30°C. The proportion of dead cells (PI stained cells) was determined using Flow cytometry (BD Calibur, BD Co., Franklin Lakes, NJ, United States). The total number of counting cells was 20,000. The minimum bactericidal concentration (MBC) is identified by determining the lowest concentration of PlnEF (when combined with 10 mM lactic acid) that totally reduces the viability of 1.0 × 10^5^CFU/mL *A. hydrophila* after 24 h under 4°C, and no colony forms on the plate after 48 h incubation at 30°C using plate counting methods.

### Lipopolysaccharide Release and Iinteraction With PlnEF Assay

The released LPS were detected by SDS-PAGE according to the previous study with little modifications (Fomsgaard et al., 1990). Briefly, the *A. hyrophila* LPL-1 cells were treated with L-lactic acid (5, 10, and 12 mM) for 0.5 to 5 h. All culture supernatants were collected, freeze-dried, and then dissolved in 100 μL of SDS-PAGE sample buffer (Novex), heated at 100°C for 10 min, and then added with proteinase K (to a final concentration of 0.25 mg/mL) and kept at 60°C for 1 h. Each sample was then evaluated by SDS-PAGE in 12% acrylamide gels; 10 μL of each sample was applied to the gel. The gels were stained with silver (0.2% AgNO_3_). The LPS released from *A. hyrophila* LPL-1 were incubated with 0, 2.5, 5, 10 μM PlnEF in sterile distilled water for 1 h at 30°C and then scanned from 190 to 210 nm using a spectrophotometer (UV2000 Unocal Shanghai instrument Co., LTD., China).

### Distribution of PlnEF on *Aeromonas hydrophila*

*Aeromonas hydrophila* cells were collected at the end of the logarithmic phase (10 h), washed twice with sterile saline, and resuspended in saline containing 10 mmol/L glucose, the final concentration of *A. hydrophila* was about 1 × 10^9^ CFU/mL (confirmed by plate count method). Part of the cells was incubated with 10 mM L-lactic acid at 4°C and collected after 2, 4, and 8 h of processing, washed twice immediately to remove the lactic acid. The rest of *A. hydrophila* cells were collected at 2, 4, and 8 h, and washed twice as the control cells. FITC-labeled PlnE and an equal amount of PlnF were added in different collected cells to a final concentration of 25 μM. About 10 μL cell suspension was dropped immediately on a clean slide, gently covered with a coverslip, and immediately observed under a laser confocal microscope (Zeiss 710 META, Germany Zeiss Company, Germany). Meanwhile, the proportion of PlnEF distributed cells (the number of cells with typical fluorescence of FITC out of the total cell number) was determined using Flow cytometry (BD Calibur, BD Co., Franklin Lakes, NJ, United States). The total number of counting cells was set as 20,000.

### Scanning and Transmission Electron Microscopy

*Aeromonas hydrophila* cells (∼1 × 10^9^ CFU/mL, confirmed by plate count method) in saline containing 10 mmol/L glucose were added with 10 mM L-lactic acid, 25 μM PlnEF, 10 mM L-lactic acid combined with 25 μM PlnEF and incubated at 30°C for 2, 4, and 8 h. The cells without PlnEF or lactic acid were set as control. Cells for scanning electron microscopy (SEM) analysis were collected by centrifugation and fixed in 2.5% glutaraldehyde at 4°C for 2-4 h. After that, the cells were dehydrated with gradient alcohol solutions and further freeze-dried. The powder of dry cells was distributed on a conductive adhesive, coated with gold, and imaged using a versatile scanning electron microscope (SEM, FEI Quanta 200, Netherlands). Part of cells treated for 8 h was fixed with 2.5% glutaraldehyde, then be prepared to ultrathin slices according to the reference (Yamanaka et al., 2005) for further observation using a transmission electron microscope (TEM, Hitachi H-7650B, Japan).

### Proteomics Analysis

#### Protein Extraction and Purification

*Aeromonas hydrophila* LPL-1 was collected by centrifugation (2,000 g, 4 min, 4°C) after 5 h incubation in LB broth and washed three times in sterile saline. The collection of bacterial cells was resuspended with (1) LB broth, (2) LB broth with 10 mM L-lactic acid, (3) LB broth with 10 mM L-lactic acid + 25 μM PlnEF, the viable count of *A. hydrophila* was equal in each sample as 1-3 × 10^9^ CFU/ml. All the samples were cultured aerobically at 30°C for 8 h and then collected by centrifugation (3,000 g, 5 min, 4°C) and washed three times with cold, sterile saline. The total protein of *A. hydrophila* cells was extracted using a Bacterial Total Protein Extraction Kit BB-3182-50T (BestBio, Shanghai, China). The extracted protein was immediately purified three times by TCA-acetone precipitation. The purified protein was dissolved in Hydration Loading Buffer I (without DTT or Bio-Lyte), and quantified by the Bradford method, and stored at −20°C for the following experiments.

#### Two-Dimensional Electrophoresis

The separation of proteins was performed by two-dimensional electrophoresis (2DE) according to the two-dimensional electrophoresis step-by-step user instructions (BioRad, Hercules, CA, United States). The specific steps are as follows: 700 μg of total protein from each sample was diluted to up to 300 μL with Hydration loading buffer I (containing Dithiothreitol (DTT) and Bio-Lyte). Each mixture was loaded onto a 17 cm precast immobilized pH gradient (IPG) gel strip (pH gradient 4-7). The first-dimension separation-isoelectric focusing (IEF)-was then carried out in the Protean IEF Cell (Bio-Rad, Hercules, CA, United States). IPG DryStrips were equilibrated in a reducing agent followed by an alkylating agent. The second dimension was performed by placing the strips on 12% acrylamide gels (Bio-Rad, Hercules, CA, United States) to allow protein separation by electrophoresis in a Criterion™ Vertical Electrophoresis Cell (Bio-Rad, Hercules, CA, United States). The analytical gels were visualized with Bio-Rad Laboratories GS-710 Calibrated Imaging Densitometer Scanner after Coomassie Brilliant Blue G-250 staining. The digitalized 2-DE gel images were studied (protein spot detection, spot matching, and semi-quantitative statistical analysis) using PDQuest 2-D Analysis Software (Bio-Rad, Hercules, CA, United States).

#### MALDI-TOF/TOF Mass Spectrometry Analysis

Spots present in only one of the conditions or displayed quantitative abundance changes of more than 1.5-fold were selected for identification by MALDI-TOF/TOF. Protein spots of interest were picked from the stained gel and were then washed and digested. The samples were mixed with a matrix solution CCA (α-cyano-4-hydroxycinnamic acid), spotted on a MALDI plate (Applied Biosystems, Foster City, CA, United States), and allowed to air-dry. To obtain a peptide mass fingerprint (PMF), lists of peak intensities and mass-to-charge (m/z) values were analyzed with a 4,800 Proteomics Analyzer MALDI-TOF/TOF Mass Spectrometer (Applied Biosystems, Foster City, CA, United States).

#### Quantitative Real-Time PCR (RT-qPCR)

RT-qPCR was performed to confirm the mRNA level of identified proteins. The reactions were prepared using TriPure reagent, 2 × SYBR Green qPCR Mix, PC48-miRNA First-strand synthesis kit (Aidlab Biotechnologies Co., Ltd., Beijing, China), according to the manufacturer’s instructions. Fifteen genes were analyzed: *acnB*, *sdhA*, *pckA*, *prpD*, *gyrB*, *gap*, *glpk*, *purA*, *rspA*, *turf1*, *turf2*, *tyrB*, *pnp*, *hptG*, *ligA*. 16S rDNA was used as a control to normalize the values. Primers for qRT-PCR were designed using Primer3Plus (Untergasser et al., 2007). The sequences of the primers are presented in Supplementary Table 1. All statistical comparisons were performed using Student’s *t*-test (*p* < 0.05).

### Statistics

All data are presented as Mean ± (SD) of 3 independent experiments. Data were analyzed using a one-way analysis of variance (ANOVA) with Dunnett’s test for comparisons to control. The SPSS 12.0 statistical software (IBM, CA, United States) was used for the analysis. *p* < 0.05 or *p* < 0.1 (two-dimensional electrophoresis) was considered significant.

## Results

### PlnEF Combined With Lactic Acid Showed Bacteriostatic and Bactericidal Activity Against *Aeromonas hydrophila* LPL-1

**FIGURE 1:**
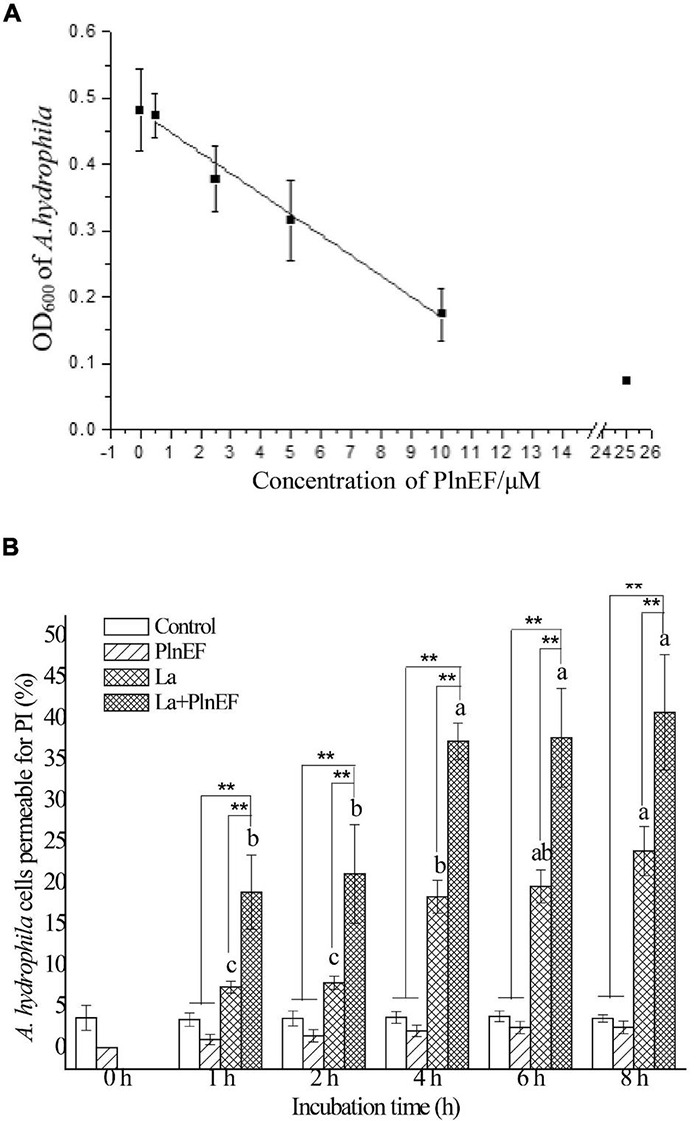
Effects of bacteriocin PlnEF and lactic acid co-treatment on inhibiting the growth and promoting lethality of *A. hydrophila* LPL-1. **(A)** *Aeromonas hydrophila* was cultured with lactic acid (10 mM) and different concentrations of PlnEF (0.5-25 μM) in 96-well cell culture plates for 12 h. The co-treatment of PlnEF and lactic acid significantly inhibited the growth of *A. hydrophila*. The results are representative of three independent experiments expressed as Means ± SD. **(B)** *Aeromonas hyrophila* cells were collected and treated with 10 mM lactic acid, 25 μM PlnEF and 10 mM lactic acid combined with 25 μM PlnEF at 30°C for 2, 4, 6, and 8 h. The co-treatment of PlnEF and lactic acid significantly promoted the proportion of dead cells. The results are representative of three independent experiments expressed as Means ± SD. Mean without a common letter indicated *p* < 0.05. Different letters of a–c indicated a significant difference between statistics of lactic acid and combined treatment at different time, ** indicated *p* < 0.01.

As shown in Figure 1, the combination of PlnEF and lactic acid showed greater bacteriostatic and bactericidal activity against *A. hydrophila* LPL-1. Introducing 10 mM lactic acid significantly increased the inhibitory activity of PlnEF against *A. hydrophila* LPL-1 (Figure 1A). In addition, with the presence of lactic acid (10 mM), the bacteriostatic activity of PlnEF showed a dose-dependent manner. The MIC of PlnEF was 25 μM that completely inhibited the growth of *A. hydrophila* LPL-1 within 12 h, shown as a low OD_600_ (0.08) as the absorption of LB broth. Besides, the co-treatment of lactic acid also improved the bactericidal activity of PlnEF against *A. hydrophila* LPL-1 (Figure 1B). The proportion of dead cells treated with 10 mM lactic acid and 25 μM PlnEF significantly increased in a time-dependent manner. After 8 h treatment, ∼40% of *A. hydrophila* LPL-1 cells were dead induced by lactic acid and PlnEF together, while only ∼20% were killed by lactic acid alone, and none were killed by PlnEF alone (The flow cytometry assay results were shown in Supplementary Figure 3). The MBC of PlnEF combined with 10 mM lactic acid against *A. hydrophila LPL-1* was 75 μM, as determined by plate counting method (as shown in Supplementary Table 2).

### Lactic Acid Caused the Release of LPS From *Aeromonas hydrophila* LPL-1 Outer Membrane

**FIGURE 2:**
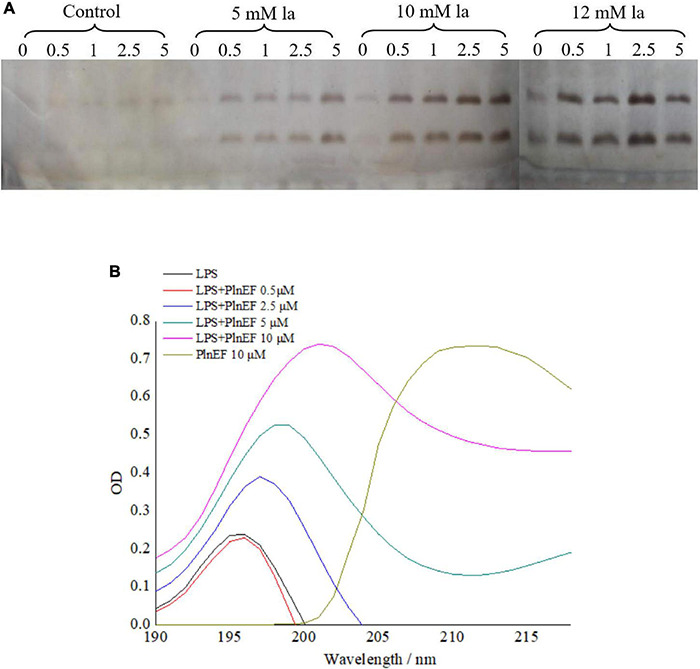
Interaction between bacteriocin PlnEF and LPS released from *A. hydrophila* LPL-1 **(A)** and effects of lactic acid on increasing LPS released from *A. hydrophila* LPL-1 **(B)**. LPS released from *A. hydrophila* LPL-1 was treated with different concentrations of PlnEF (0.5, 2.5, 5, and 10 μM) showed a red shift in absorption peak. *Aeromonas hyrophila* LPL-1 cells were treated with different concentrations of lactic acid (5, 10, and 12 mM) for 0, 0.5, 1, 2.5, and 5 h. The release of LPS from *A. hyrophila* LPL-1 significantly increased by lactic acid in a dose-dependent and time-dependent manner.

The release of LPS by lactic acid and the interaction between LPS with PlnEF was investigated in *A. hydrophila* LPL-1. In our study, lactic acid treatment (5, 10, 12 mM) significantly induced the LPS release from *A. hydrophila* LPL-1 (Figure 2A). With the increase of concentration and incubation time, a significant increase of released LPS was observed, indicating a stimulatory effect of lactic acid on LPS release. The released LPS showed a characteristic absorption peak at 195 ~196 nm, while a significant red shift was observed after PlnEF treatment, suggesting a binding between PlnEF and LPS (Figure 2B). Notably, the peak values increased in a concentration-dependent manner of PlnEF, indicating an interaction between LPS and PlnEF.

### Pre-Treatment With Lactic Acid Allowed the Accumulation of PlnEF on/in *Aeromonas hydrophila* LPL-1 Cells

**FIGURE 3:**
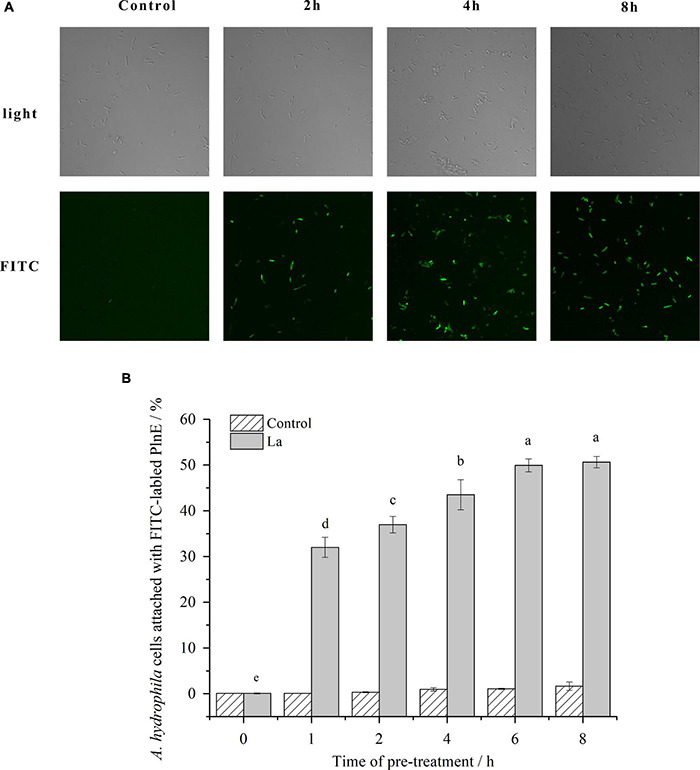
Effects of lactic acid on promoting the accumulation of FITC labeled PlnEF in *A. hydophila* LPL-1. *Aeromonas hyrophila* LPL-1 cells were treated with FITC labeled PlnEF and lactic acid (25 μM) for 2, 4, and 8h. The co-treatment significantly increased the number of FITC labeled PlnEF in *A. hyrophila* LPL-1. **(A)** The FITC positive cells under a laser confocal microscope. **(B)** The proportions of FITC-positive cells in *A. hyrophila* LPL-1. The results are representative of three independent experiments expressed as Means ± SD. Means without a common letter are significantly different (*p* < 0.05).

The penetration and accumulation of PlnEF on/in *A. hydrophila* LPL-1 cells were measured with laser confocal microscopy. Without lactic acid pre-treatment, there was no FITC-labeled PlnE observed on/in *A. hydrophila* LPL-1 (Figure 3A). In comparison, there was an obvious accumulation of green fluorescence produced by FITC-labeled PlnE on/in *A. hydrophila* LPL-1 cells after pre-treated with lactic acid. Besides, the proportion of the green-fluorescent cells increased with the time prolongation of lactic acid pre-treatment (Figure 3A). The results of flow cytometry further showed a precise increasing proportion of FITC-positive cells (Figure 3B). After 6 h pre-treating with 10 mM lactic acid, up to 50% of the bacterial cells showed the fluorescent signal of FITC-labeled PlnE (Figure 3B). These results suggested that pre-treatment with lactic acid allowed the accumulation of PlnEF on/in *A. hydrophila* LPL-1 cells.

### PlnEF Combined With Lactic Acid-Induced Significant Damage and Deterioration of *Aeromonas hydrophila* LPL-1 Cellular Structure

**FIGURE 4:**
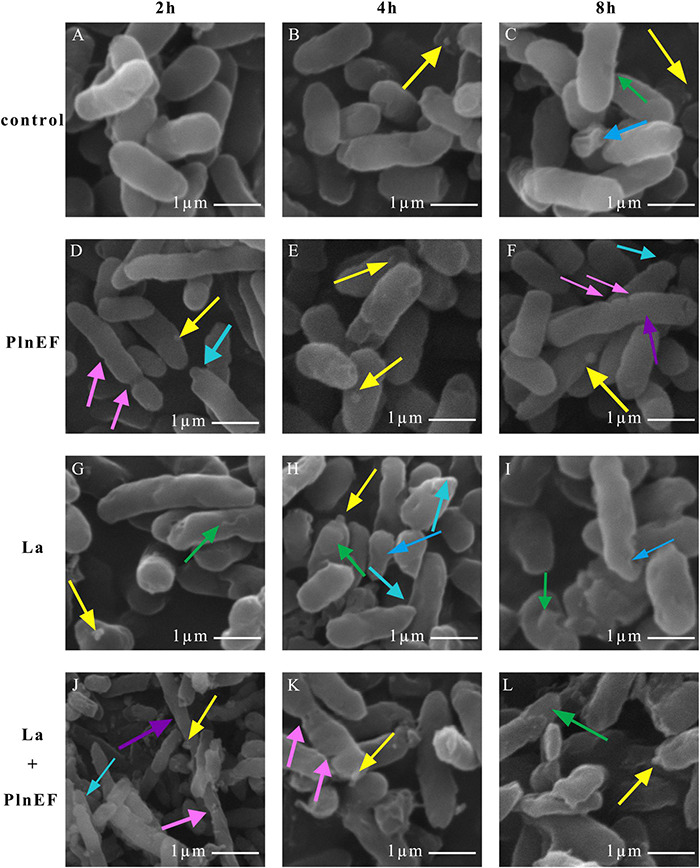
Effects of bacteriocin PlnEF and lactic acid co-treatment on changing cell morphology of *A. hyrophila* LPL-1. *Aeromonas hyrophila* LPL-1 cells were treated with lactic acid (10 mM) and/or PlnEF (25 μM) at 4°C for 2, 4, and 8 h. Images were observed using a scanning electron microscope (SEM). The scale bar indicates length 1 μm, HV = 15-20 kV, direct mag (20,000-50,000). **(A–C)** the control cells, **(D–F)** the PlnEF treated cells, **(G–I)** lactic acid treated cells, **(J–L)** PlnEF combined lactic acid treated cells. Lactic acid treatment induced outer membrane damage (green arrows), deformation and shrinkage (blue arrows), and apical surface protrusion (cyan arrow). The co-treatment of PlnEF and lactic acid induced extra small vesicles (yellow arrows), multiple splitting points (pink arrows), holes (red arrows), increase in length (purple arrows) and fragmentation of cracking bacteria (orange arrows).

**FIGURE 5:**
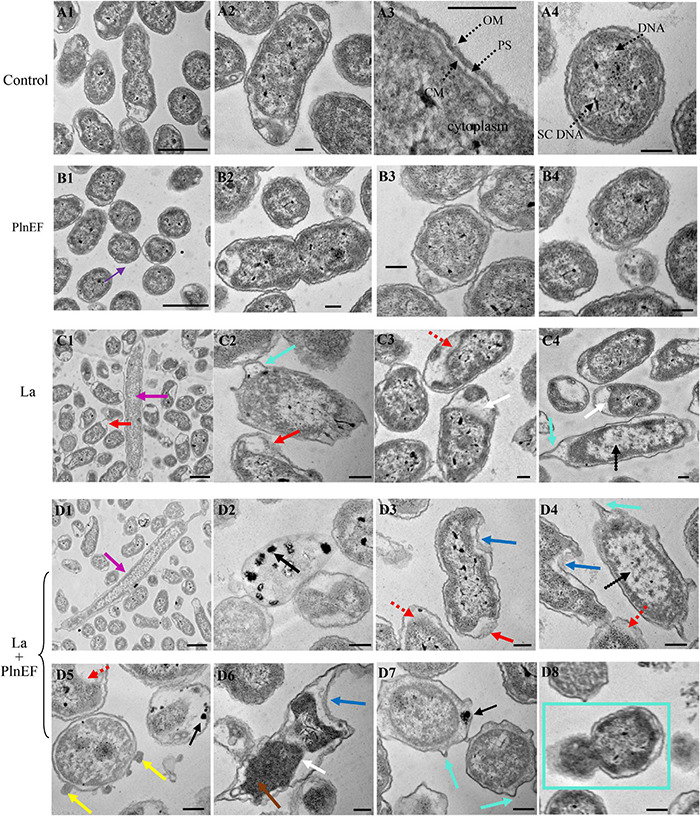
Effects of bacteriocin PlnEF and lactic acid co-treatment on affecting internal structural deformation of *A. hydrophila*. *Aeromonas hyrophila* LPL-1 cells were treated lactic acid (10 mM) and/or PlnEF (25 μM) at 4°C for 8 h. Images were observed using a Hitachi H-7650B transmission electron microscope (TEM). The scale bar indicates length 1 μm, HV = 80 kV, direct mag (20,000-1,00,000). **(A1–A4)** the control cells, **(B1–B4)** the PlnEF treated cells, **(C1–C4)** lactic acid treated cells, **(D1–D8)** PlnEF and lactic acid combined treated cells. The binary fission (BF), outer membranes (OM), cytoplasmic membrane (CM), periplasmic space (PS), supercoiled DNA (SCDNA) are visible. Lactic acid treatment induced protrusion (cyan arrows), en (purple arrows), outer membrane damage (red solid line arrows), cell inner membranes damaged (red dotted arrows), the outer and inner membrane separation (white arrows), and reduced electron density region (black dotted arrows). Co-treatment of PlnEF and lactic acid induced extra dark granules (black solid line arrows), small vesicles (yellow arrows), deepening (brown arrows), protruding part (cyan box), sag (blue arrows).

The effect of PlnEF and lactic acid either alone or in combination on cell morphological and structural change of *A. hydrophila* LPL-1 was studied using scanning electron microscopy (SEM) (Figure 4) and transmission electron microscopy (TEM) (Figure 5). Under the normal cultural condition, the control cells of *A. hydrophila* LPL-1 appeared as rod shapes with a blunt circle at both ends, with a smooth surface and intact morphology (Figure 4A). With increasing incubation time, moderate outer membrane damage (green arrows), cellular deformation, and shrinkage (blue arrow) were observed in a small portion of bacterial cells (Figure 4C). Meanwhile, some nanometer vesicles (yellow arrows) appeared on the surface of *A. hydrophila* LPL-1 control cells (Figure 4B). *A. hydrophila* LPL-1 treated with PlnEF alone had a similar morphology with the control cells (Figures 4D–E). During 8 h incubation with PlnEF, most of *A. hydrophila* LPL-1 cells remained typical smooth surface and rod shape with a few nanometer vesicles around (Figure 4E). However, the length of the *A. hydrophila* LPL-1 cells increased (purple arrows) when cultured with PlnEF compared against the control, and several cells had two suspected splitting points (pink arrows) (Figures 4D,F). Besides, apical surface protrusion (cyan arrow) was detected on a small proportion of bacterial cells (Figures 4D,F). Compared to PlnEF treatment alone, lactic acid treatment alone induced severer shrinkage (blue arrow) and apical surface protrusion (cyan arrow), and outer membrane damage (green arrow) in *A. hydrophila* LPL-1 cells (Figures 4H–J), indicating a strong disruption effect of lactic acid on the cell morphology. The strongest disruption was found when *A. hydrophila* LPL-1 was treated with lactic acid and PlnEF together. Serious surface deformation, shrinkage, collapse was observed in *A. hydrophila* LPL-1 cells (Figures 4K,L). Some of the cells even had visible holes (red arrow), and visible fragments of cracking bacteria (orange arrow). Furthermore, the combination of lactic acid and PlnEF also caused multiple splits (pink arrow) in several cells, and more nanometer vesicles (yellow arrow) appeared on the surface of the cells after 2 h treatment. Corresponding changes were found in the internal structures of *A. hydrophila* LPL-1 (Figure 5). The control cells of *A. hydrophila* LPL-1 were rod-shaped in the longitudinal section and elliptical-shaped in the cross-section (Figures 5A1–A4). All cells had clear edges of outer membranes (OM), cytoplasmic membrane (CM), and uniform periplasmic space (PS, the inner space between OM and CM). The cytoplasm was evenly distributed, shown as unanimous electron density. The DNA was distributed randomly in the cell, with some dark filamentous and dots in the middle of the DNA, possibly a supercoiled DNA (SCDNA). The PlnEF treated cells did not show any noticeable change in inter-structure compared to the control sample, indicating a limited effect of PlnEF on *A. hydrophila* LPL-1 (Figures 5B1–B4). Lactic acid-treated cells had visible deformation, such as irregular protrusion (cyan arrows), and lengthen (purple arrows) as well as evident outer membrane damage (red solid line arrows) (Figures 5C1–C4). Besides, a separation of the outer and inner membrane (white arrow) was observed. Moreover, the area of DNA was brighter (red dashed-line arrow) than control, and the content of the high electron density substance of DNA remarkably decreased. Similar to the SEM results, *A. hydrophila* LPL-1 treated with lactic acid combined with PlnEF showed more severe deformation on the inner structure (Figures 5D1–D8). The separation of the outer and inner membrane (white arrows) was observed. Cell inner membranes damaged (red dotted arrows) showing an incomplete and blurred shape. Protruding vesicles (yellow arrows) were captured. Cytoplasm loss was indicated by decreased electron density in most of the cells. An abnormal cytoplasm condense was revealed by deepening color through the center of the cell (brown arrows). Some cells were elongated (purple arrows). Besides, individual cells inflated at damaged parts of the outer membrane, forming a protruding part (cyan box) without the outer membrane’s surroundings. There were electron condensed particles, shown as dark granules (black solid line arrows), distributed between isolated cell walls and cell membranes.

### The Combination of Lactic Acid and PlnEF Leads to an Alternation of Proteomic Profile in *Aeromonas hydrophila* LPL-1.

**FIGURE 6:**
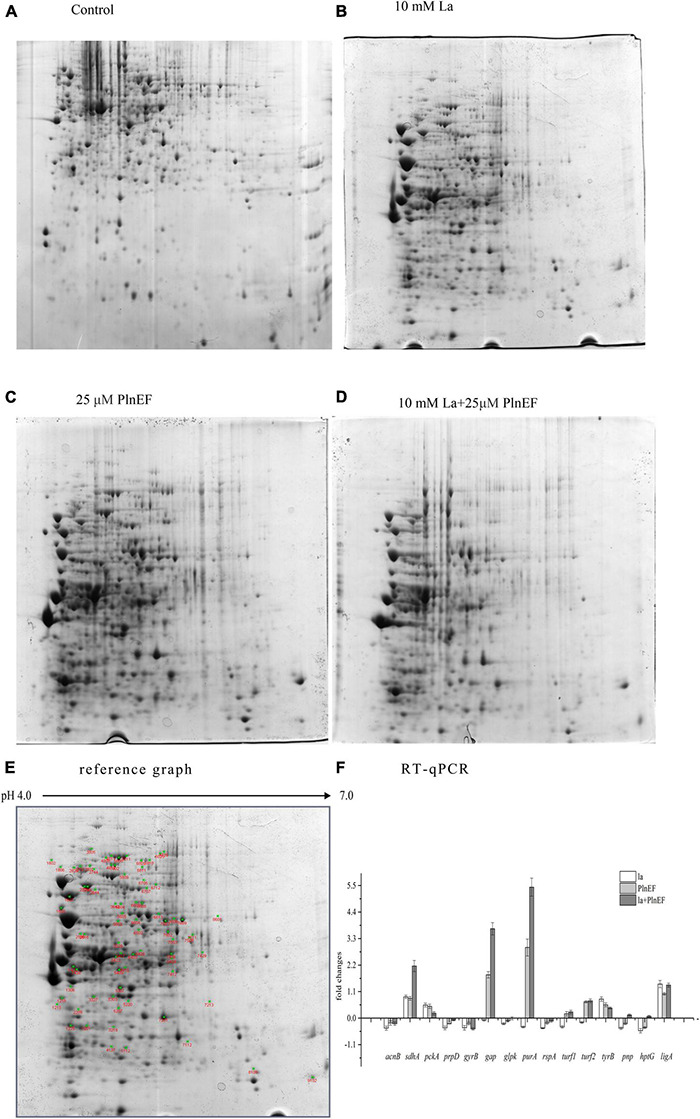
Effects of bacteriocin PlnEF and lactic acid co-treatment on the proteomic profile of *A. hydrophila*. **(A–D)** A two-dimensional electrophoresis (2-DE) separation of total proteins from *A. hydrophila*. The cells of *A. hydrophila* LPL-1 (1–3 × 10^9^ CFU/ml) were cultured in LB broth (control), LB broth with 25 μM PlnEF; 10 mM L-lactic acid; and 10 mM L-lactic acid + 25μM PlnEF aerobically at 30°C for 8 h. The first dimension comprised an 17-cm non-linear pH 4–7 immobilized pH gradient (IPG) subjected to isoelectric focusing. The second dimension was a 21-cm 12% SDS-PAGE (sodium dodecyl sulfate polyacrylamide gel electrophoresis) gel. Proteins were detected by Coomassie Brilliant Blue G-250 staining. The non-linear pH range of the first-dimension IPG strip is indicated along the top of the gel, acidic pH to the left. The Mr (relative molecular mass) scale can estimate the molecular weights of the separated proteins**. (E)** Two-dimensional polyacrylamide gel electrophoresis (PAGE) reference map of the whole proteins from *A. hydrophila* LPL-1. Using the PDQuest software (Bio-Rad), the average gel of each population was compared with the reference map gel to identify the differentially expressed protein spots (green cross). **(F)** The relative mRNA levels of several genes in *A. hydrophila* LPL-1 treated by la-10mM lactic acid, PlnEF-25 μM PlnEF, la + EF-10 mM lactic acid + 25 μM PlnEF for 8 h revealed by Quantitative RT-PCR. acnB: bifunctional aconitate hydratase 2/2-methylisocitrate; sdhA: succinate dehydrogenase flavoprotein subunit; pckA: phosphoenolpyruvate carboxykinase; prpD: 2-methylcitrate dehydratase; gap: glyceraldehyde-3-phosphate dehydrogenase (GAPDH); glpk: glycerol kinase (GK); purA: adenylosuccinate synthetase (ADSS); rspA: 30S ribosomal subunit protein S1; turf1, turf2: elongation factor Tu; aromatic amino acid aminotransferase (AAA-ATs) gyrB: DNA gyrase subunit B; ligA. NAD-dependent DNA ligase; tyrB: threonyl-tRNA synthetase; pnp: polynucleotide phosphorylase/polyadenylase; hptG: heat shock protein 90.

A two-dimensional electrophoresis (2-DE) separation of total proteins, using the same concentrations of prepared proteins from *A. hydrophila* cells cultured in LB broth, and treated with lactic acid, plantaricin E/F either solely or in combination, were shown in Figures 6A–D. The differentially expressed protein spots based on the comparison of the control sample were pointed out in the reference map Figure 6E. The number of over-expressed protein numbers was 3, 12, and 8 for single PlnEF, single lactic acid, and their combination treatment, respectively. The down-expressed number of protein was 5, 49, and 30 in turn (Supplementary Tables 3–5). We identified 27 differentially expressed proteins in *A. hydrophila* cells (Data are available via ProteomeXchange with identifier PXD029702), and further tested the results by q-PCR (Figure 6F). The relative mRNA level of *acnB*, *prpD*, *glpk*, *turf*1/2 in the combined treated sample was consistent with the protein level. However, there was a discrepancy between mRNA and protein abundance of *tyrB*, *pnp*, *htpG* and *ligA*. The differently expressed proteins participated in several pathways (Table 1), including energy metabolism (TCA, glycolysis, pyruvate metabolism, gluconeogenesis, glycerophospholipid synthesis), amino and protein metabolism, purine, and pyrimidine metabolism, DNA replication, transcription and repair, peptide transport, and stress response. In terms of energy metabolism, AcnB and ADSS were significantly (*p* < 0.1) over-expressed, GAPDH was significantly (*p* < 0.1) down-expressed in the single lactic acid-treated sample. In the combined treated sample, there was a significant (*p* < 0.1) down-expression of AcnB, PrpD, and GK, suggesting an inhibition in energy metabolism by the combined treatment. In terms of protein synthesis, in the single lactic acid-treated sample, the levels of two detected elongation factor Tu (5,422 and 7,407) were significantly down-regulated, but the level of point 5,438 that also stands for elongation factor Tu significantly increased; the aromatic amino acid aminotransferase and threonyl-tRNA synthetase decreased statistically (*p* < 0.1). In combined treated samples, the levels of elongation factor Tu, threonyl-tRNA synthetase (5,422 and 5,438), and threonyl-tRNA synthetase were significantly (*p* < 0.1) higher than the control group. As for nucleotide synthesis, both single lactic acid treatment and combined treatment significantly (*p* < 0.1) reduced the level of polynucleotide phosphorylase/polyadenylase. Interestingly, single lactic acid treatment reduced the content of DNA gyrase subunit B, but combined treatment increased its level. The combined treatment also reduced the level of NAD-dependent DNA ligase. Hsp90 (heat shock protein 90) is a chaperone protein that assists other proteins in folding correctly. Combined treatment significantly (*p* < 0.1) reduced the level of heat shock protein 90, indicating a potential interference in protein folding and function.

**TABLE 1:** The differently expressed proteins of *A. hydrophila* LPL-1 treated with lactic acid and PlnEF combined with lactic acid against control cells.

Protein number	Fold change			Protein name	Pathway	Gene	Gene ID
	La/Con	Com/Con	Com/La				
5811	1.95*	0.2*	0.1	bifunctional aconitate hydratase 2/2-methylisocitrate (AcnB)	TCA	*acnB*	gi| 492595947
6608	0.47	1.22	2.61*	succinate dehydrogenase flavoprotein subunit (SdhA)	TCA	*sdhA*	gi| 511289116
6613	0.63	1.5	2.38*	phosphoenolpyruvate carboxykinase (PEPCK)	glycolysis,TCA,pyruvate metabolism	*pckA*	gi| 491452275
7511	0.52	0.1*	0.2*	2-methylcitrate dehydratase (PrpD)	pyruvate metabolism	*prpD*	gi| 507519522
7201	0.54*	1.06	1.95*	glyceraldehyde-3-phosphate dehydrogenase (GAPDH)	glycolysis, gluconeogenesis	*gap*	gi| 491477967
7503	1.11	0.34*	0.31*	glycerol kinase (GK)	glycerophospholipid synthesis	*glpk*	gi| 507521596
1432	1.54*	0.49	0.32*	adenylosuccinate synthetase (ADSS)	purine metabolism, amino acid metabolism	*purA*	gi| 511291306
1607	0.39	1.38	3.52*	30S ribosomal subunit protein S1	protein synthesis	*rpsA*	gi| 507523578
5422	0.21*	1.42*	6.79*	elongation factor Tu	protein synthesis	*tuf1/tuf2*	gi| 117617738
5438	2.01*	2.66*	1.33	elongation factor Tu	protein synthesis	*tuf1/tuf2*	gi| 117617738
7408	0.45*	1.39	3.11*	elongation factor Tu	protein synthesis	*tuf1/tuf2*	gi| 498318947
7412	0.17*	0.54	3.12*	aromatic amino acid aminotransferase (AAA-ATs)	Aromatic amino acid synthesis	*tyrB*	gi| 498360645
6707	0.27*	2.83*	10.36	threonyl-tRNA synthetase	threonyl-tRNA synthesis	-	gi| 511290436
1218	0.55*	0.15*	0.28*	polynucleotide phosphorylase/polyadenylase	Purine metabolism, pyrimidine metabolism	*pnp*	gi| 511291449
6403	0.16*	1.08	6.81*	polynucleotide phosphorylase/polyadenylase	Purine metabolism, pyrimidine metabolism	*pnp*	gi| 657056548
6818	0.2*	2.24*	11.05*	DNA gyrase subunit B	DNA replication and transcription	*gyrB*	gi| 511291783
8601	1.02	0.41*	0.40*	NAD-dependent DNA ligase	DNA replication and repair	*ligA*	gi| 640508216
7609	0.74	1.14	1.54*	peptide ABC transporter periplasmic peptide-binding	peptide transport	-	gi| 117617738
3705	1.23	0.44*	0.35*	heat shock protein 90	Stress response	*htpG*	gi| 516376089
6426	–	–	2.35*	hypothetical protein	–	–	gi| 511289064
2644	1.52*	0.33*	0.22*	hypothetical protein	–	–	gi| 330830010
2209	3.86*	1.94*	0.5*	hypothetical protein	–	–	gi| 511290703

*P < 0.1.

## Discussion

Lactic acid and bacteriocin are two important metabolites of lactic acid bacteria, which have been reported for their antimicrobial activities (Barbosa et al., 2017; Komesu et al., 2017; Mokoena, 2017; Gao et al., 2019; Vieco-Saiz et al., 2019). However, there is less study on the synergistic inhibitory mechanism of bacteriocin and lactic acid. The present study investigated the synergistic inhibitory activity and mechanism of IIb bacteriocin PlnEF and lactic acid on potential Gram-negative pathogen *A. hydrophila* LPL-1.

In the present study, we found that combining class IIb bacteriocin-PlnEF with lactic acid significantly enhanced the inhibition ability against potential Gram-negative pathogen *A. hydrophila* LPL-1. Besides, we also found PlnEF and lactic acid had synergistic inhibition against several Gram-negative pathogens. Thus the inhibition activity against Gram-negative pathogen of PlnEF may be universal but not specific against *A. hydrophila* LPL-1 (Supplementary Figure 4). The result was an essential addition to the universal synergistic action of lactic acid and different bacteriocins, given that the synergistic inhibitory effects were confirmed between class I bacteriocin-nisin (Nykänen et al., 1998), class IIa pediocin AcH (Kalchayanand et al., 1992), and pediocin PA-1 (Wang et al., 2020). In addition, the PlnEF are cationic peptides and effective in micromolar-level. According to our previous study, the MIC (minimum inhibitory concentration) and the MBC (minimum bactericidal concentration) of PlnEF against *Lactiplantibacillus plantarum* (former *Lactobacillus plantarum*) were 8 μM and 16 μM, respectively (Zhang et al., 2016). In this study, the MIC and MBC of PlnEF against *A. hydrophila* were 25 and 75 μM when combined with 10 mM lactic acid. These results suggested a comparable but lower antibacterial efficiency of PlnEF against *A. hydrophila* to Gram-positive bacteria when lactic acid was incorporated. This is similar to our previous work, where the MIC of class IIa bacteriocin pediocin PA-1 in combination with lactic acid against *A. hydrophila* ATCC 35654 (50 μM) and CICC 10500 (30 μM) was also higher than its MIC (5 μM) against a sensitive Gram-positive bacteria *L. plantarum* (Wang et al., 2020).

The outer membrane (OM) of *A. hydrophila* works as an efficient permeability barrier to protect them against bacteriocin, such as PlnEF. L-lactic acid has been reported to be an efficient OM permeabilizer (Vaara, 1992), as well as exert the activity to released LPS from Gram-negative bacteria, such as *Salmonella enterica* serovar Typhimurium (Alakomi et al., 2000) and *A. hydrophila* ATCC 35654 (Wang et al., 2020). Our study observed the release of LPS by lactic acid in *A. hydrophila* LPL-1. Besides, the interaction, most likely the electrostatic interaction between PlnEF and LPS was found in this study, indicating the protective effect of LPS against bacteriocin in Gram-negative bacteria. However, with the treatment of lactic acid, the LPS barrier was damaged. And further transportation and accumulation of FITC-labeled PlnEF in *A. hydrophila* were revealed in the present study. Generally, to exert biological activity, antimicrobial peptides are required to interact and/or communicate with cells first (Hancock and Rozek, 2002). As PlnEF shows hydrophobic properties, there is a high possibility that the specific location of PlnEF is in the inner membrane by directly sticking into the membrane, which is believed to be the primary mechanism of membrane-leaking bacteriocin (Nissen-Meyer et al., 2009; Moghal et al., 2020). The loss of membrane integrity caused by the combination of PlnEF and lactic acid shown by SEM and TEM supports the idea that PlnEF anchor and cause injuries on the inner membrane. A similar result has been reported in our previous research on the synergistic inhibitory effect of lactic acid and class IIa bacteriocin pediocin PA-1 against *A. hydrophila* (Wang et al., 2020). As reported, class IIb bacteriocin used UppP (undecaprenyl pyrophosphate phosphatase) (Kjos et al., 2014), streptococcal membrane protein LsrS (Biswas and Biswas, 2014), or a Zn-dependent metallopeptidase (Uzelac et al., 2013) as their receptors. However, either the location of PlnEF or PlnEF-associated receptor was not identified in the present study yet. Further studies are needed to determine the receptor in *A. hydrophila* for PlnEF.

The *A. hydrophila* cells treated with lactic acid and PlnEF also exhibited morphological and size changes in our study. It has been claimed that environmental stress (Typas et al., 2012; Mueller and Levin, 2020), or substance interfering dividing related genes that affect the bacteria size and/or morphology (Domínguez-Cuevas et al., 2013; Vedyaykin et al., 2019; Di Somma et al., 2020). Combined treatment with PlnEF and lactic acid caused stress to cells, which could further affect the expression of genes and proteins related to the cell size and morphology. Besides, the PlnEF and lactic acid-treated cells showed a lot of vesicles, which were similar to the outer membrane vesicles (OMVs) reported from other studies, such as *E. coli* treated with sericin (Xue et al., 2016). Gram-negative bacteria could produce such OMVs, consisting of protein, lipid, and lipopolysaccharide enclosed by a lipid bilayer (Jan, 2017; Avila-Calderón et al., 2021), during growth or under pressure (Schwechheimer and Kuehn, 2015; Toyofuku et al., 2019). Our previous research also found OMVs induced by the synergistic treatment of lactic acid and class IIa bacteriocin pediocin PA-1 in *A. hydrophila* (Wang et al., 2020). These OMVs are believed to be toxic to other organisms and benefit the survival of their producing bacteria (Avila-Calderón et al., 2015). Supportively, we also found reduced cytotoxicity when *A. hydrophila* was treated with PlnEF and lactic acid (unpublished data). However, the involvement of OMVs and cytotoxicity reduction remains unclear. DNA is the most important molecule for cellular activity. In our study, the original DNA region became electron-light after treated with lactic acid, indicating an abnormal change in DNA conformation. This will result in a failure of cell division or other DNA-involved cellular activity. The lightening of electron density in the DNA region had been reported in *A. hydrophila* treated with lactic acid and PA-1 (Wang et al., 2020), *Escherichia coli* and *Salmonella* cells treated with lactic acid (Wang et al., 2015), *Cronobacter sakazakii* treated with *Chrysanthemum buds* crude extract (Chang et al., 2021), *E. coli* cells treated with polyhexamethylene (Zhou et al., 2010). Meanwhile, apparent condensation granules, supposed to be condensed DNA or protein, were visible in cells treated with lactic acid and PlnEF together in our study.

These results indicated a complicated and cooperative mechanism between PlnEF and lactic acid to inhibit *A. hydrophila*. As vesicles induction and loss of membrane integrity were typical in PlnEF sensitive cells after being treated with PlnEF (Zhang et al., 2016), we believe that one possible mechanism of PlnEF against *A. hydrophila*, under the assistance of lactic acid, was membrane disruption. Moreover, the treatments induced condense of cytoplasm, relaxed DNA, and failure of dividing might be an inspiring mechanism that contributes to the synergistic effect of PlnEF and lactic acid. The complex cellular morphological changes suggested that in addition to the enhanced OM permeabilizing action of PlnEF induced by lactic acid, the presence of PlnEF could stimulate the activity of lactic acid in return.

To further elucidate the mechanism involved in the inhibition of *A. hydrophila*, we had analyzed the differentially expressed proteins aroused by co-treatment of PlnEF and lactic acid. We found significant down-regulation of key proteins in TCA, suggesting inhibition of energy metabolism. Hotshock protein Hsp90 was down-regulated, but DNA gyrase GraB was up-regulated by co-treatment of PlnEF and lactic acid, suggesting abnormal protein folding and DNA status, which was in accordance with the emerging of outer membrane vesicles and reduced DNA electron density proved in SEM and TEM.

**FIGURE 7:**
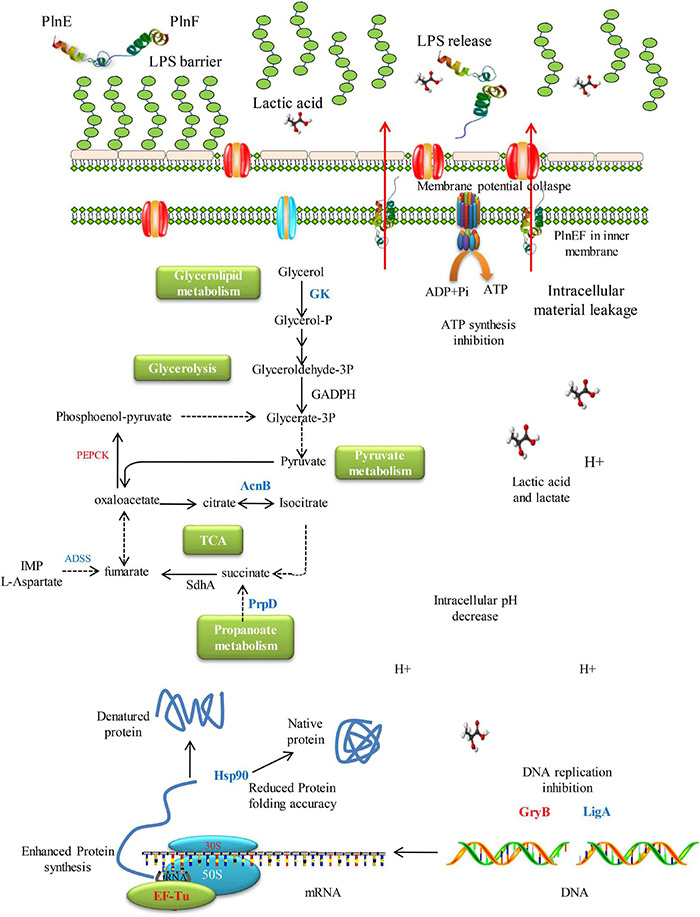
Schematic mechanism of inhibition of synergistic inhibition of PlnEF and lactic acid on *A. hydrophila.* The LPS works as a barrier against PlnEF. After the release of LPS induced by lactic acid, PlnEF insert into the inner membrane of *A. hydrophila*, causing the collapse of membrane potential. In the center of the cell, reduction of energy metabolism, down-regulation of Hsp90 but up-regulation of DNA gyrase GraB, suggesting abnormal protein folding and DNA status, which was in accordance with the outer membrane vesicles and reduced DNA electron density proved in SEM and TEM.

In conclusion, the present study investigated the synergistic bacteriostatic and bactericidal activity of bacteriocin PlnEF and lactic acid against potential Gram-negative pathogen, *A. hydrophila* LPL-1. The overall mechanism of the synergistic activity of PlnEF and lactic acid against *A. hydrophila* was shown in Figure 7. The LPS is acting as a barrier against PlnEF. Upon the release of LPS as induced by lactic acid, PlnEF integrates and causes damage in the inner membrane of *A. hydrophila*. In the center of the cell, there is a reduction of energy metabolism, down-regulation of Hsp90, and up-regulation of DNA gyrase GraB, indicating abnormal protein folding and DNA status, consistent with the outer membrane vesicles and reduced DNA electron density shown in SEM and TEM. Thereby, lactic acid and PlnEF synergistically inhibit bacterial growth and cause cell death.

## Data Availability Statement

The whole-genome sequencing data of *A. hydrophila* LPL-1 are deposited in GenBank, accession number PRJNA767215. The proteome profiling data are deposited in ProteomeXchange, accession number PXD029702.

## Author Contributions

All authors listed have made a substantial, direct, and intellectual contribution to the work, and approved it for publication.

## Conflict of Interest

The authors declare that the research was conducted in the absence of any commercial or financial relationships that could be construed as a potential conflict of interest.

## Publisher’s Note

All claims expressed in this article are solely those of the authors and do not necessarily represent those of their affiliated organizations, or those of the publisher, the editors and the reviewers. Any product that may be evaluated in this article, or claim that may be made by its manufacturer, is not guaranteed or endorsed by the publisher.

## Footnotes

1
http://www.ncbi.nlm.nih.gov/bioproject/767215
