# Supplementary material for: Synergistic Inhibition of Plantaricin E/F and Lactic Acid Against Aeromonas hydrophila LPL-1 Reveals the Novel Potential of Class IIb Bacteriocin
Source: Front Microbiol. 2022 Feb 15;13:774184. doi: 10.3389/fmicb.2022.774184 (PMC8886044; doi:10.3389/fmicb.2022.774184)
Supplement: Supplementary file 1 [file Data_Sheet_1.pdf]

Supplementary Table 1 Primers used in Real-time PCR for differentially expressed protein

Gene name	Primer sequence	Gene name	Primer sequence
pnp	F: tctacggtcacgagcagatg<br>R: gtaggcttcacccagctcag	*purA*	F: aagtactggtcgagcgcatt<br>R: tgaattcgaaggtcgggtag
acnB	F: ctcccataccctgctgatgt<br>R: agatcgtcggtgttggtttc	*htpG*	F: ggagacggtcaccttgttgt<br>R: aaacacggcctgaagctcta
gyrB	F: acgagtacaacccggacaag<br>R: agaaggtcagcagcagggta	*ligA*	F: gcgtggtctacaaggtggat<br>R: ggaactcgacgttctcaagc
gap	F: acaccaacgaccagaacctc<br>R: ctgctgcagataggcattga	*rpsA*	F: tggaccaacaagaacatcca<br>R: ttggatttgcactgcttcag
tuf1	F: atgtacggaacgcctacctg<br>R: acaccgctacccgtcactac	*sdhA*	F: tgcacaccctctatcagcag<br>R: gatgtgaccgtcctggttct
tuf2	F: cagtgcagaaccacggacta<br>R: caggtaggcgttccgtacat	*pckA*	F: ccgatgagctggatcttctc<br>R: gcgatgaagagctgaaaacc
glpk	F: ctcttccgagctcttccaga<br>R: ggaatccatcgcctatcaga	*tyrB*	F: ggagttgctgacgaagaagc<br>R: ttcatggacatcgcctatca
prpD	F: tcgcccttattaccatgagc<br>R: cttgaacagcacgttttcca		

Supplementary Table 2 The minimum bactericidal concentration (MBC) of PlnEF combined with lactic acid determined by plate counting method

Growth of colony		PlnEF levels / μM							
		0	50	55	60	65	70	75	80
lactic acid levels /mM	0	+	+	+	+	+	+	+	+
	10	+	+	+	+	+	+	-	-

Note: + represent growth of a colony, - represent no growth of a colony.

Supplementary Table 3 The differently expressed proteins in 10 mM L-lactic acid-treated *A.hydrophila* LPL-1 compared against the control cells

Protein number	Fold change	Protein number	Fold change	Protein number	Fold change
4718	0.05	1802	0.32	1016	4.3
5906	0.07	7105	0.33	2209	3.86
7508	0.07	7515	0.33	7104	3.44
7706	0.12	1806	0.34	11102	2.83
3642	0.14	7612	0.34	2307	2.78
9102	0.14	6805	0.35	4808	2.37
7428	0.15	3810	0.37	2506	2.28
6403	0.16	6809	0.41	3201	2.25
6706	0.16	7408	0.45	5301	1.78
5605	0.17	5409	0.48	1208	1.73
7412	0.17	7422	0.48	2823	1.72
6818	0.2	6813	0.49	3327	1.51
2641	0.24	2737	0.5		
6712	0.24	8109	0.5		
8103	0.24	7606	0.51		
6604	0.26	5307	0.53		
6709	0.26	7201	0.54		
6811	0.26	1215	0.55		
6707	0.27	1218	0.55		
4807	0.29	6603	0.57		
6825	0.29	1438	0.61		
7426	0.29	5603	0.62		
3826	0.3	7806	0.64		
4811	0.3	7603	0.66		
5604	0.3				

Supplementary Table 4 The differently expressed proteins in PlnEF treated *A.hydrophila* LPL-1 compared against the control cells

Protein number	Fold change	Protein number	Fold change
3809	0.22	501	2.14
4505	0.51	8303	3.38
5503	0.67	602	1.85
8005	0.6		
8401	0.7		

Supplementary Table 5 The differently expressed proteins in PlnEF combined with lactic acid-treated *A.hydrophila* LPL-1 compared against the control cells

Protein number	Fold change	Protein number	Fold change	Protein number	Fold change
7428	0.03	6106	0.27	7660	6.35
1215	0.04	4812	0.3	1133	3.96
5807	0.07	5241	0.3	4137	3.32
7511	0.1	2644	0.33	5416	2.66
3106	0.12	5810	0.33	2823	2.63
1218	0.15	7503	0.34	2647	2.53
7615	0.15	5207	0.36	2209	1.94
6711	0.16	5303	0.47	4522	1.63
2516	0.17	1605	0.51		
5806	0.17	5605	0.51		
5802	0.2	5603	0.52		
5811	0.2	6612	0.53		
4907	0.22	7706	0.54		
2737	0.24	5307	0.56		
6154	0.24	1701	0.63		
