# Supplementary figures and images for: Synergistic Inhibition of Plantaricin E/F and Lactic Acid Against Aeromonas hydrophila LPL-1 Reveals the Novel Potential of Class IIb Bacteriocin
Source: Front Microbiol. 2022 Feb 15;13:774184. doi: 10.3389/fmicb.2022.774184 (PMC8886044; doi:10.3389/fmicb.2022.774184)

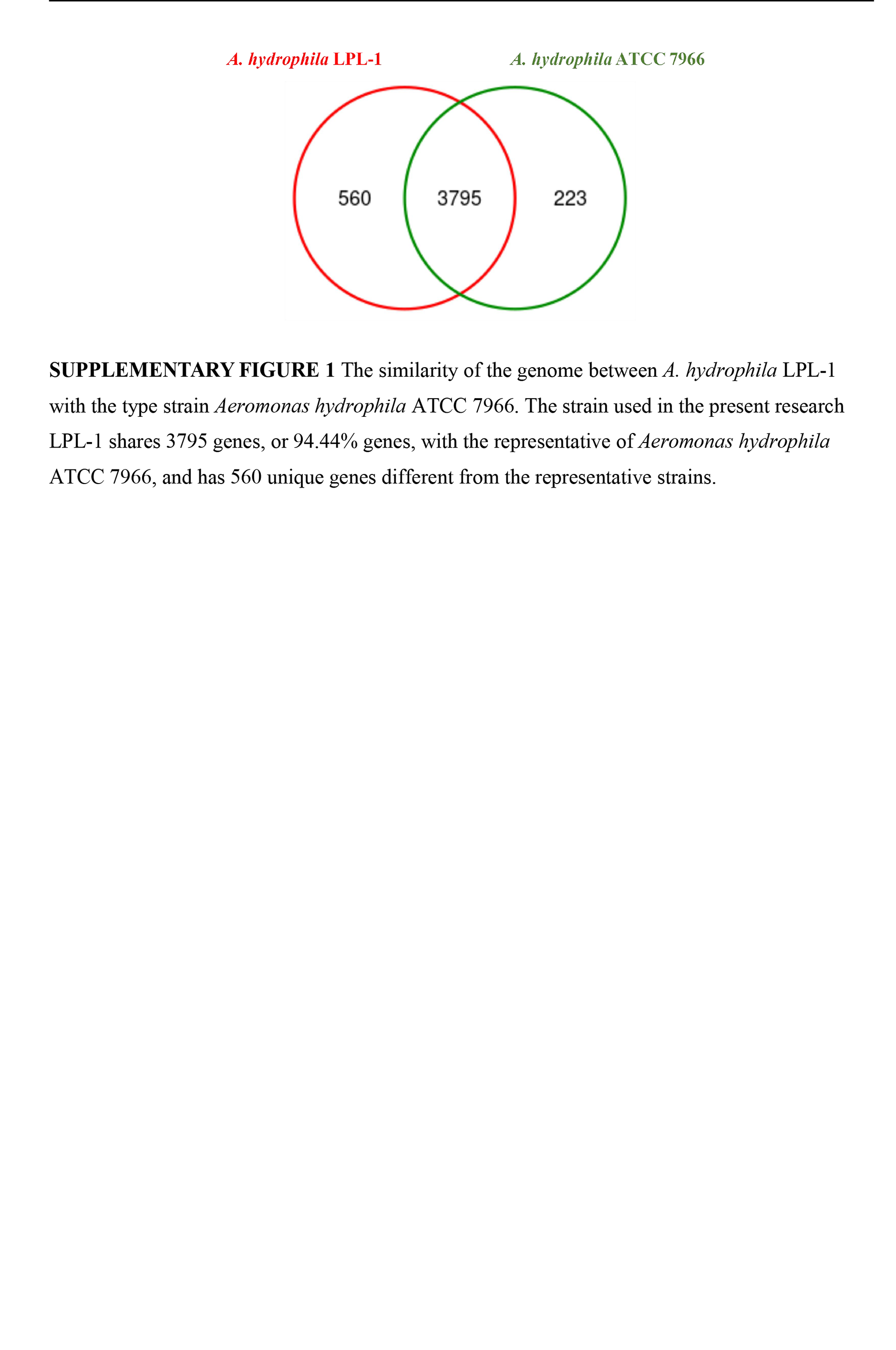

Supplement: Supplementary file 2 [file Image_1.tif]

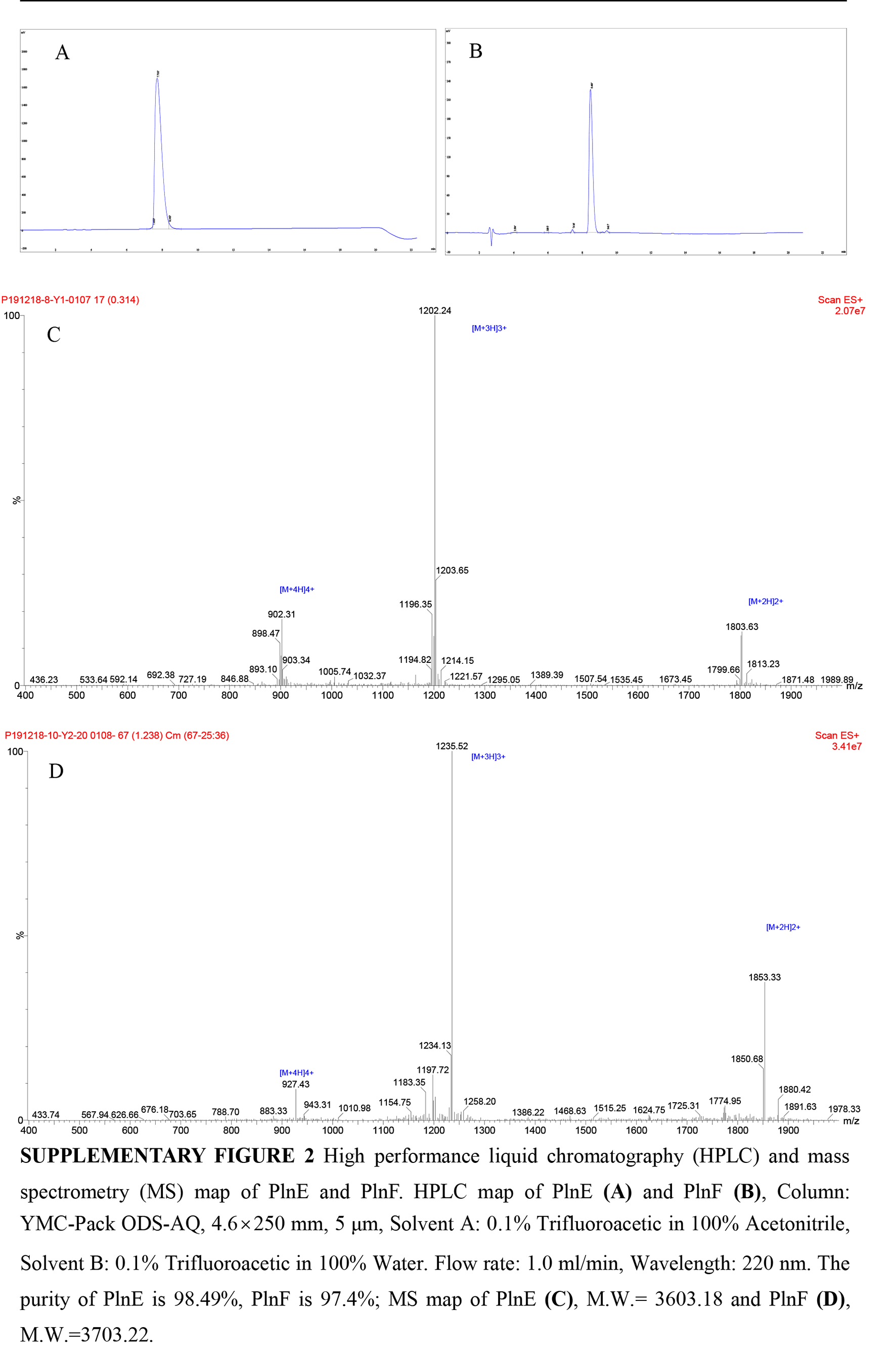

Supplement: Supplementary file 3 [file Image_2.tif]

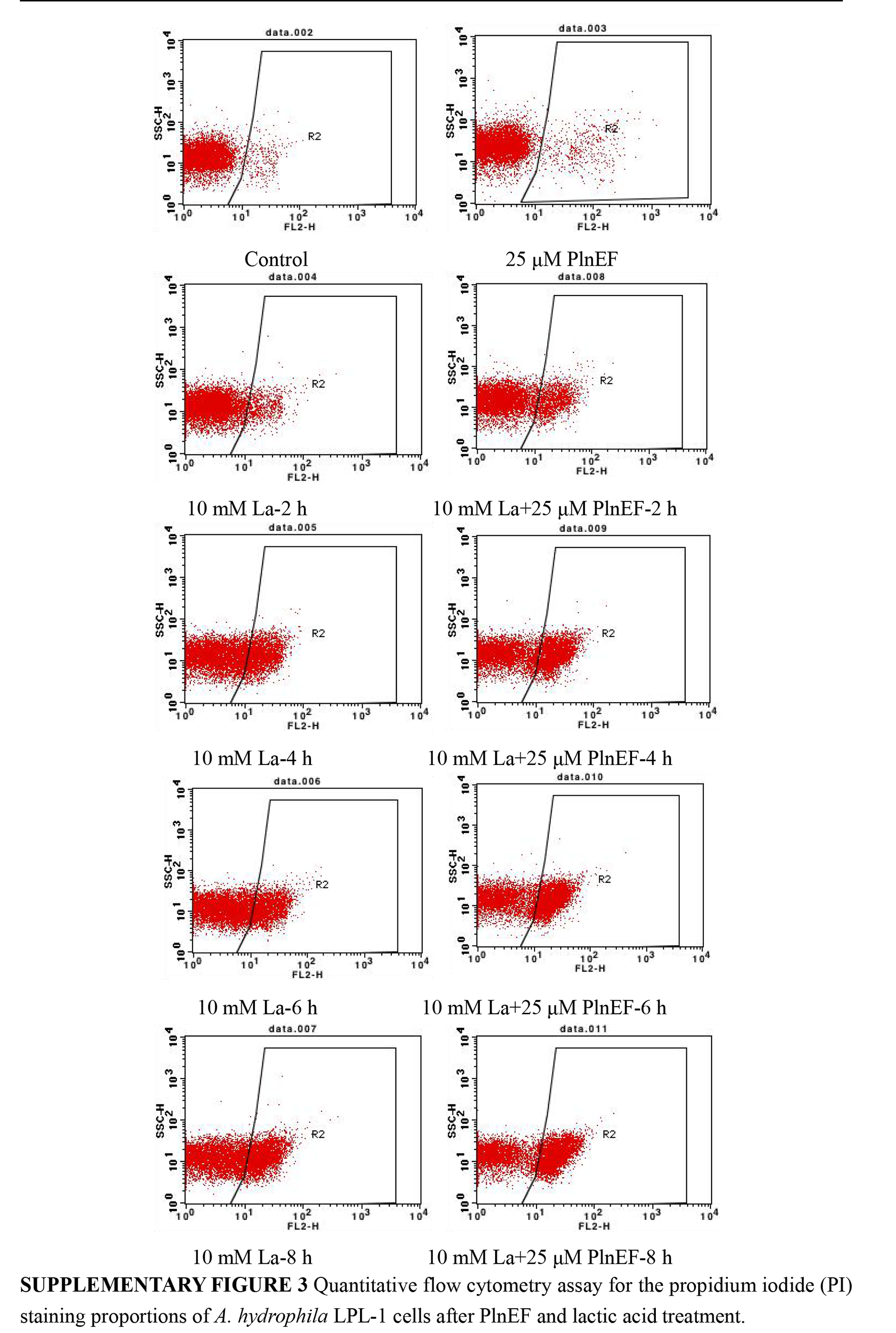

Supplement: Supplementary file 4 [file Image_3.tif]

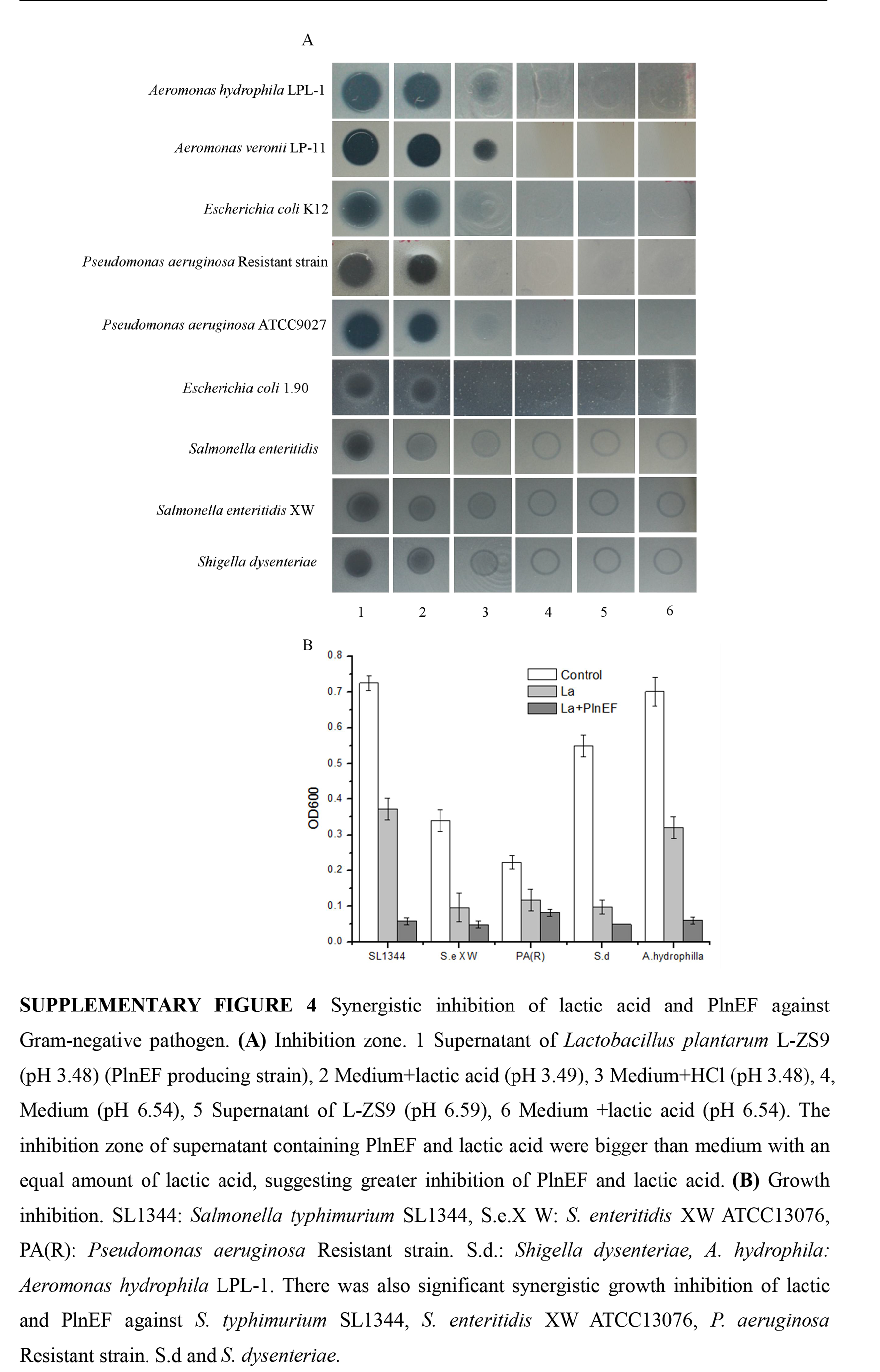

Supplement: Supplementary file 5 [file Image_4.tif]
